# Supplementary material for: Semisynthetic Cardenolides Acting as Antiviral Inhibitors of Influenza A Virus Replication by Preventing Polymerase Complex Formation
Source: Molecules. 2020 Oct 21;25(20):4853. doi: 10.3390/molecules25204853 (PMC7587960; doi:10.3390/molecules25204853)

SUPPLEMENTARY MATERIAL

Semisynthetic cardenolides acting as antiviral inhibitors of influenza A virus replication by preventing polymerase complex formation

Laurita Boff [a,b]; André Schreiber [a]; Aline da Rocha Matos [a,c]; Juliana Del Sarto [a,d]; Linda Brunotte [a]; Jennifer Munkert [e]; Flaviano Melo Ottoni [d]; Gabriela Silva Ramos [d]; Wolfgang Kreis [e]; Fernão Castro Braga [d]; Ricardo José Alves [d]; Rodrigo Maia de Pádua [d]; Cláudia Maria Oliveira Simões [b,*], Stephan Ludwig [a]

[a] Institute of Virology (IVM), Centre for Molecular Biology of Inflammation (ZMBE), Westfaelische Wilhelms University (WWU), Münster, Germany

[b] Laboratório de Virologia Aplicada, Programa de Pós-Graduação em Farmácia, Universidade Federal de Santa Catarina (UFSC), Florianópolis, SC, Brazil.

[c] Laboratório de Vírus Respiratórios e do Sarampo, National Influenza Center (NIC)/ World Health Organization (WHO), Instituto Oswaldo Cruz/Fiocruz, Rio de Janeiro, RJ, Brazil.

[d] Departamento de Produtos Farmacêuticos, Faculdade de Farmácia, Universidade Federal de Minas Gerais (UFMG), Belo Horizonte, MG, Brazil.

[e] Pharmaceutical Biology, Department of Biology, Friedrich-Alexander-Universitity Erlangen-Nuremberg, Germany.

[*] Corresponding author:

E-mail address: *claudia.simoes@ufsc.br* (C. M. O. Simões)

Supplementary data index

1. Fig. S1…………………………………..……………………………………………….02
2. Fig. S2…...…………………………………………………………………….……..03

Fig. S1. Antiviral activity of new cardenolide derivatives **C7c**, **C10**, **C11** and **C12** and digitoxigenin against distinct Influenza A and B Virus strains, at 1 µM. ** p < 0.01, *** p < 0.001 and **** p < 0.0001 *vs* DMSO, one-way ANOVA, post-hoc test Dunnet.

A) Influenza A Virus [Panama/2007/1999 (H3N2)] on A549 and MDCK cells.

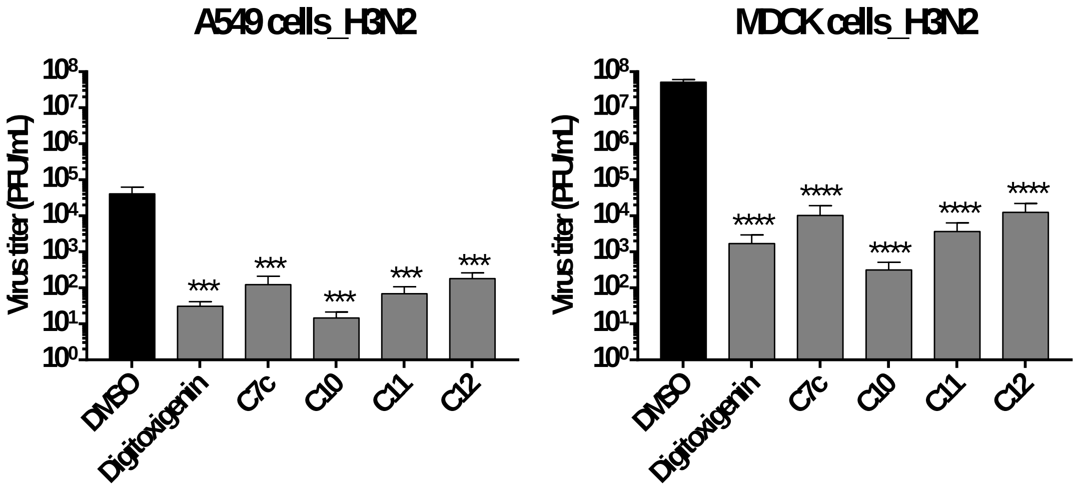

B) Influenza A Virus [Hamburg/04/2009(H1N1pdm)] on A549 and MDCK cells.

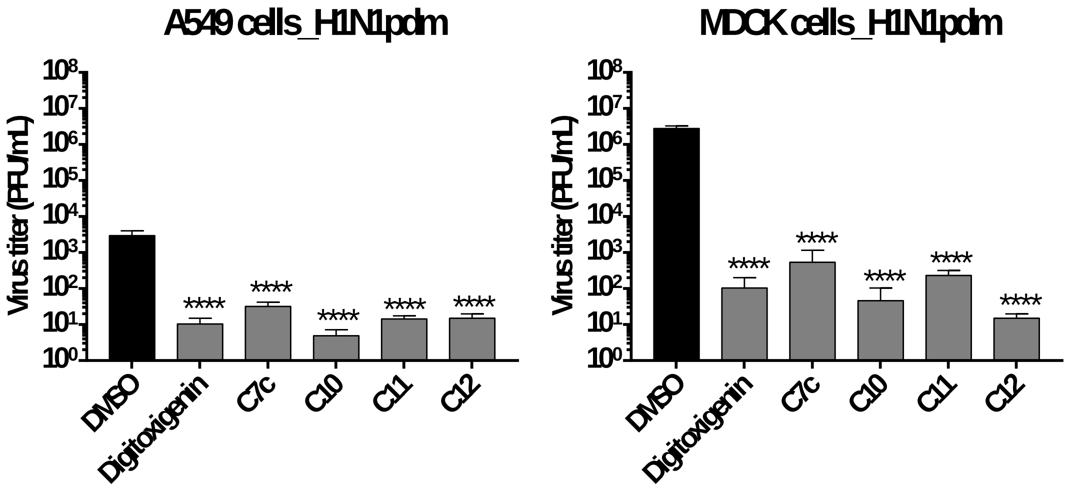

C) Influenza B Virus (Lee/40) on MDCK cells.

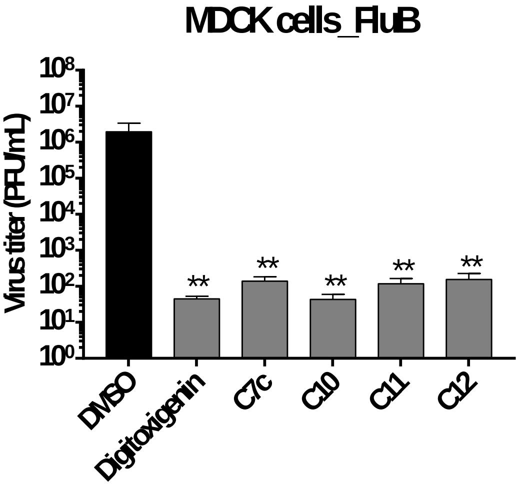

D) Influenza A Virus [Seal/Mass/1-SC35M/80 (H7N7) (SC35M)] on A549 and MDCK cells.

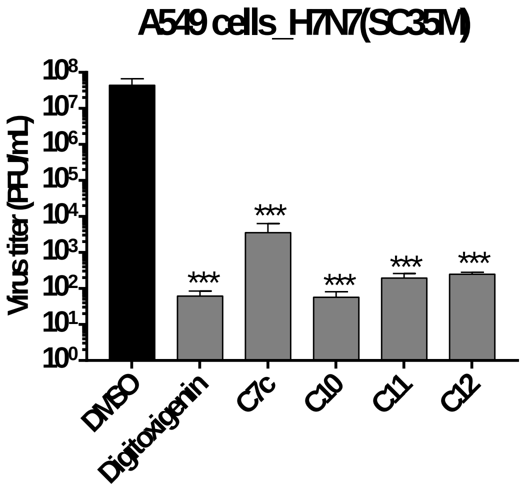

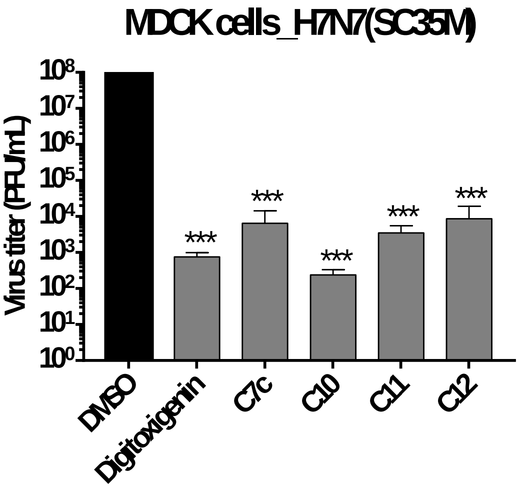

Fig. S2. Viability of human tumor-free lung explants after treatment with **C10** and **C11**. Lung tissue explants were treated and their viability over time was assessed by measuring LDH release in culture supernatants, which were collected at the indicated time points. Results are presented as Optical Densities at 450 nm and have been corrected according to the individual weights of the tissue fragments (mg). The experiment was repeated with lung tissues derived from four independent donors. Each time point represents mean (± SEM). DMSO served as solvent control. * $p < 0.05$ *vs* MOCK, one-way ANOVA, post-hoc test Dunnet.

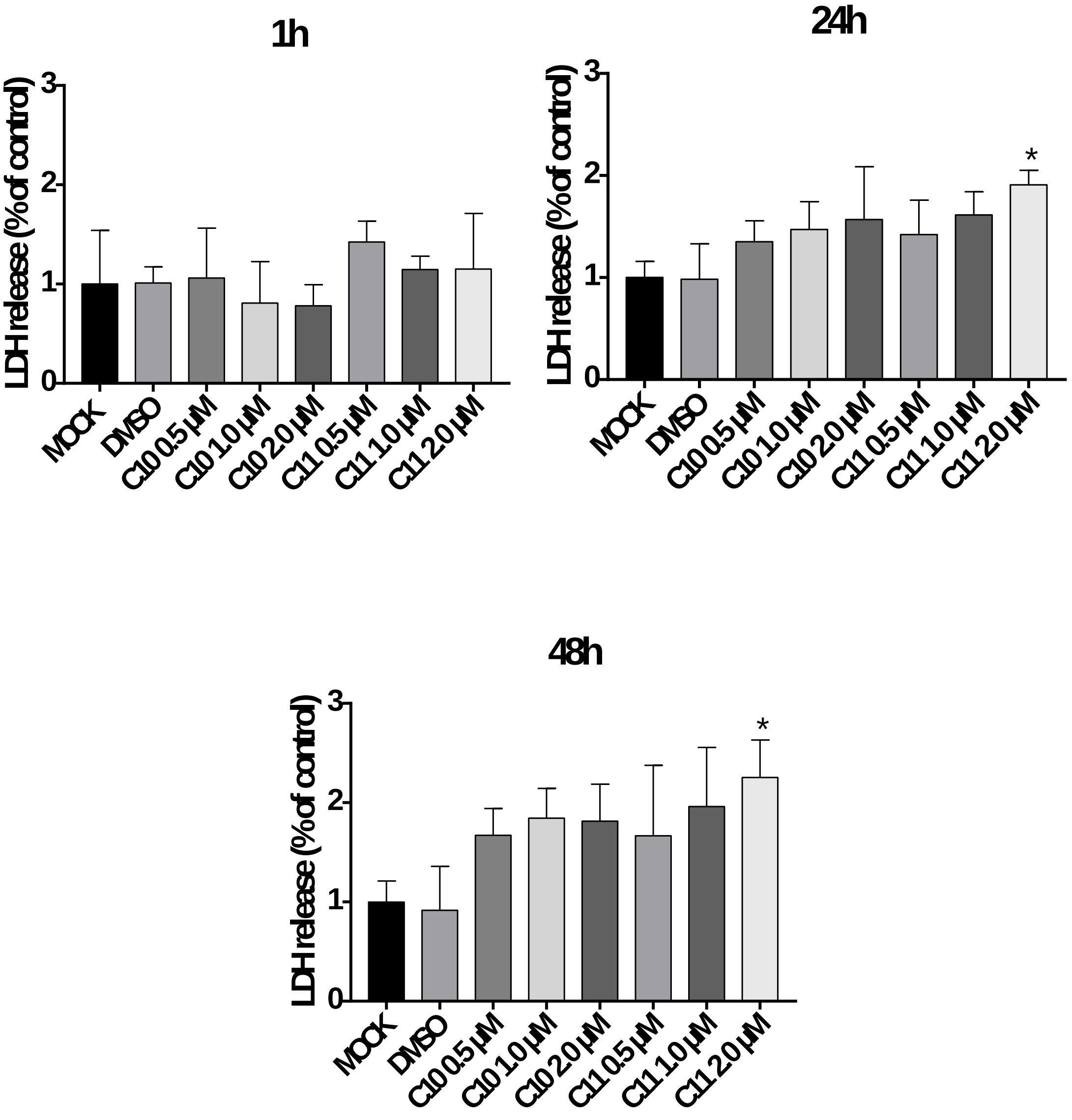

Supplement: Supplementary file 1 [file molecules-25-04853-s001.pdf]
